# Prognostic value of systemic immune-inflammation index in the diagnosis of preeclampsia

**DOI:** 10.1016/j.heliyon.2024.e28181

**Published:** 2024-03-19

**Authors:** Mucahit Kapci, Kemal Sener, Adem Cakir, Ertugrul Altug, Ramazan Guven, Akkan Avci

**Affiliations:** a Department of Emergence Medicine, Republic of Turkey, Ministry of Healthy Başaksehir Çam and Sakura State Hospital, Istanbul, Turkey; b Department of Emergence Medicine, Republic of Turkey, Mersin State Training and Research Hospital Hospital, Mersin, Turkey; c Department of Emergency Medicine, Ministry of Health of Turkey, Canakkale Mehmet Akif Ersoy State Hospital, Canakkale, Turkey; d Health Science University, Adana City Research and Training Hospital, Department of Emergency Medicine, Adana, Turkey

**Keywords:** Preeclampsia, Systemic immune-inflammation index, Platelet count, Neutrophil-to-lymphocyte ratio

## Abstract

**Background:**

Preeclampsia is a serious complication of pregnancy with negative consequences for the mother and fetus. It was aimed to investigate whether the systemic immune inflammation index is a parameter that will facilitate the diagnosis of preeclampsia.

**Methods:**

This retrospective and single-center study included patients diagnosed with preeclampsia after admission to the emergency department and those who met the inclusion criteria. Vital parameters, demographic data, medical history, white blood cell count, platelet count, neutrophil count, systemic immune-inflammation index values, biochemical parameters, and gestational weeks were analyzed in each patient.

**Results:**

A total of 40 patients with preeclampsia (preeclampsia group) and 40 normal pregnant women (control group) were included. Laboratory tests revealed that the mean WBC, neutrophil, and lymphocyte counts were significantly higher in the preeclampsia group than in the control group, whereas the preeclampsia group had a significantly lower mean platelet count than the control group (p < 0.001). The sensitivity and specificity for the cut-off value of 758.39 × 10^9^/L systemic immune-inflammation index in pregnant patients with preeclampsia was 77.5% and 67.5%, respectively (AUC: 0.705; 95% CI: 0.587–0.823; **p = 0.002**). No significant difference was observed between the mean neutrophil-to-lymphocyte ratio in preeclampsia diagnosis.

**Conclusion:**

The systemic immune-inflammation index may be used as a marker to help in establishing the diagnosis of preeclampsia. We believe that this index is an important prognostic indicator because it concurrently evaluates neutrophil and lymphocyte values—which indicate the inflammation process—and platelet count, i.e., an indicator of coagulopathy.

## 1 Introduction

Preeclampsia is a serious complication that occurs during pregnancy and it with negatively affects the mother and fetus. It is one of the leading causes of maternal and infant mortality, and it is observed in 2%–8% of pregnancies worldwide [1]. Globally, preeclampsia was reported to cause 14% of maternal deaths [2]. The American College of Obstetrics and Gynecology (ACOG) defines preeclampsia as the presence of hypertension and proteinuria after 20 weeks of gestation in a previously normotensive patient [3]. In cases of severe preeclampsia, maternal end-organ damage, systemic abnormalities (hemolysis, thrombocytopenia, elevated liver enzymes), and intrauterine growth retardation may occur [4].

A successful semi-allograft reaction occurs during the development of a normal pregnancy. The appropriate inflammatory state of the maternal (maternal–fetal interface) and systemic immune system induces blastocyte implantation, which consequently triggers trophoblast cell proliferation, differentiation, and infiltration and promotes placental growth and development. In patients with preeclampsia, an overactive inflammatory response of abnormal maternal immune tolerance is observed to accept the fetal semi-allograft. This inflammatory response was reportedly caused by cytokines released from white blood cells (WBCs). To elaborate, neutrophils can release a variety of inflammatory cytokines to activate inflammatory cells and immune responses, leading to oxidative stress and endothelial damage and eventually promoting preeclampsia progression. Therefore, lymphocyte, neutrophil, and monocyte counts and their ratios have been thoroughly explored in the literature [[5], [6], [7]]. The contact of platelets with the developing endothelial damage area activates the coagulation system. Pregnant women typically experience increased platelet production and consumption. The increased platelet consumption is attributable to abnormalities in the coagulation system and platelet activation and the occurrence of thrombocytopenia, which may be used as an important sign of preeclampsia [[8], [9], [10]].

The systemic immune-inflammation index (SIII) is a novel systemic inflammatory prognostic indicator used in many diseases and clinical conditions as an indicator of inflammation, particularly in relation to prognosis in patients with malignancies. An association between SIII and many chronic/acute inflammatory diseases has been reported [11]. SIII may be a parameter that facilitates preeclampsia diagnosis because it is easy and inexpensive to perform and use for calculation and can be calculated with only complete blood count test parameters, does not contain any subjective value and makes an evaluation of neutrophils along with lymphocytes and platelets.

Peripheral blood cells are easy to measure and are increasingly used because they are accessible. Leukocyte, lymphocyte, platelet, and monocyte counts can be measured from blood cell count and neutrophil-to-lymphocyte ratio (NLR), monocyte-to-lymphocyte ratio, mean platelet volume can be easily calculated. SIII is an index used especially in patients with malignancies and has been frequently used in the literature for the diagnosis and follow-up of acute inflammatory processes. We think that the evaluation of the inflammatory process and the change in platelet counts in patients with preeclampsia may have diagnostic value and be considered a prognostic marker. In our literature review, there is very limited information about SIII in patients with preeclampsia. In addition, it is the first study in the literature comparing the SIII values of hypertensive pregnant patients with healthy pregnancies and preeclamptic pregnant patients. We think that our study will contribute to the limited data in the literature.

## 2 Methods

### 2.1 Study setting

This study was initiated after obtaining approval from the ethics committee (Ethics committee no: KAEK/2023.06.980). Our study was designed as a retrospective and single-center study. The study was performed with patients diagnosed with preeclampsia after admission to the emergency department from February 15, 2021, to February 15, 2023, and patients who met the inclusion criteria. Vital parameters, demographic data, medical history, WBC count, platelet count, neutrophil count, SIII values, biochemical parameters, and gestational weeks of the patients were obtained from the hospital automation system (Hospital Information Management System-HIMS) and recorded on a preformed study form.

Pregnant women with hypertension who presented to the emergency department, aged ≥18 years, and whose complete data were available were included in the study. Out of these patients, patients under the age of 18 years, patients with missing data, patients whose outcome could not be followed up and whose medical history was unknown, patients with a diagnosis of malignancy in their medical history, history of hematologic disease, bone marrow pathology, anti-inflammatory or immunosuppressive drug use, and patients with a focus of infection were excluded from the study. The study included 40 patients diagnosed with preeclampsia (preeclampsia group) and 40 hypertensive women with normal pregnancy (control group).

The preeclampsia group was defined as pregnant patients who developed hypertension with proteinuria or end organ damage after the 20th week of pregnancy. The definitive diagnosis of preeclampsia was made by an obstetrics and gynecology specialist. Patients who met the diagnostic criteria but were not diagnosed with preeclampsia by an obstetrics and gynecology specialist were excluded from the study. Normal pregnant women; they were selected among pregnant women who completed their pregnancies in a healthy manner and were followed up due to hypertension during their pregnancy and did not experience any fetal or maternal health problems during their follow-up.

Excluded patients included 2 patients excluded due to history of malignancy, 12 patients due to incomplete data, 5 patients due to history of hematologic disease, 5 patients due to presence of a focus of infection, and 4 patients due to inconsistent blood pressure measurements, out of 108 patients identified by searching the files and automation system (Hospital Information Management System).

### 2.2 Data calculation

In the current study, calculations of the cases were made from the results obtained from the cases. P, N and L stand for peripheral platelet, neutrophil and lymphocyte counts, respectively. NLR (N/L Ratio) and SIII ((P × N)/L ratio) values were calculated [11].

### 2.3 Simple size calculation

Power analysis was done with G power 3.9. Since the power analysis in our study will be done with the control group, it was calculated as a *t*-test. For power analysis, a power of 0.80 was calculated with a 95% confidence interval and an error rate of alpha = 0.05. Since there was no study similar to our study, the estimated rate was accepted as 0.5. As a result of this calculation, 38 patients, at least 19 for both the study and control groups, were determined. In our study, a total of 80 (40 + 40) patients were included in the study and control groups as a precaution case of missing dates.

### 2.4 Statistical analysis

Data were analyzed with SPSS Package Program version 24.0. The descriptive data were presented as number, percentage, mean, standard deviation (SD), median, minimum, and maximum values. The distribution normality of the data was analyzed using the Kolmogorov–Smirnov Test. In univariate analysis, normally distributed continuous variables were expressed as mean ± SD and compared using T-Test. Pearson Chi-Square Test was used to analyze categorical variables. Fisher's Exact Test was used in the presence of less than 5 variables in categorical variables. T-Test was used for comparison of two independent numerical data. Diagnostic accuracy was assessed using ROC (receiver operating characteristic) curve analysis. Appropriate cut-off values were determined and sensitivity and specificity values were calculated for parameters with area under the curve (AUC) above 0.600. Logistic regression analysis was performed to investigate the effects of parameters in predicting preeclampsia in the cases.

P < 0.05 was accepted as the level of statistical significance.

## 3 Results

Our study was performed with 40 patients with preeclampsia and 40 women with normal pregnancy. The mean age was 28.98 ± 7.14 years in the preeclampsia group and 30.10 ± 5.82 years in the control group, and no significant intergroup difference was observed. Vital parameters showed that systolic and diastolic blood pressure were significantly higher and mean saturation was significantly lower in the preeclampsia group; however, there was no significant difference between the two groups in terms of mean pulse rate (Table 1).

**Table 1:** Comparison of demographic, clinical, and laboratory data of the patient and control groups.

Parameter	Preeclampsia Group (n = 40) Mean ± SD	Control Group (n = 40) Mean ± SD	p
**Demographic Data**			
Age (years)	28.98 ± 7.14	30.10 ± 5.82	0.442
**Vital Data**			
Systolic BP	145.45 ± 22.22	122.80 ± 11.98	**<0.001**
Diastolic BP	91.20 ± 10.62	69.520 ± 8.93	**<0.001**
Pulse	93.18 ± 14.81	91.68 ± 18.07	0.686
Saturation	97.25 ± 1.34	98.30 ± 0.80	**<0.001**
**Laboratory Tests**			
WBC ( × 10^9^/L)	17.22 ± 5.30	11.97 ± 3.36	**<0.001**
Hgb (mg/dL)	11.72 ± 1.50	11.63 ± 1.52	0.793
Neutrophils ( × 10^9^/L)	12.96 ± 5.57	8.93 ± 3.43	**<0.001**
Lymphocytes ( × 10^9^/L)	3.25 ± 1.14	2.04 ± 1.10	**<0.001**
Platelets ( × 10^9^/L)	191.55 ± 62.76	255.60 ± 69.29	**<0.001**
CRP (mg/dL)	18.26 ± 7.62	17.80 ± 14.70	0.916
Urea	19.93 ± 5.70	25.58 ± 10.65	**0.004**
Creatinine	0.61 ± 0.12	0.71 ± 0.14	**0.021**
ALT	19.08 ± 14.73	41.40 ± 75.41	0.070
AST	31.38 ± 18.25	81.95 ± 142.10	**0.028**
**Proportions**			
SIII ( × 10^9^/L)	944.23 ± 861.12	1600.37 ± 1486.27	**0.018**
NLR	4.81 ± 3.65	6.46 ± 5.79	0.131
**Other Parameters**			
Gestation Week	34.67 ± 5.18	34.63 ± 4.28	0.963
Number of Pregnancy	2.28 ± 1.77	2.45 ± 1.77	0.659
Length of Hospital Stay	5.05 ± 3.52	7.98 ± 6.69	**0.017**

*: Pearson χ^2^ Test; **: T-Test was used.

Sd: standard deviation; CRP: C Reactive Protein; NLR: Neutrophil-to-lymphocyte ratio; PLR: Platelet-to-lymphocyte ratio; PNR: Platelet-to-neutrophil ratio; LNR: Lymphocyte-to-neutrophil ratio; SIII: Systemic immune-inflammatory index.

The laboratory tests revealed that the preeclampsia group had higher mean WBC, neutrophil, and lymphocyte counts than the control group, whereas the preeclampsia group had significantly lower mean levels of platelets, urea, creatinine, and AST than the control group. No significant intergroup difference was observed in terms of the mean values of hemoglobin, CRP, and ALT (Table 1).

Examination of the mean NLR and systemic immune-inflammation index (SIII) calculated using the ratio of laboratory parameters of the cases demonstrated that the mean SIII was significantly lower in preeclampsia group than in the control group. However, no significant intergroup difference was noted in terms of mean NLR (Table 1).

Gestational week, number of pregnancies, and duration of hospitalization were compared. Although the hospitalization duration was significantly higher in the preeclampsia group, no significant difference was observed between the two groups in terms of gestational week and number of pregnancies (Table 1).

**Fig. 1:**
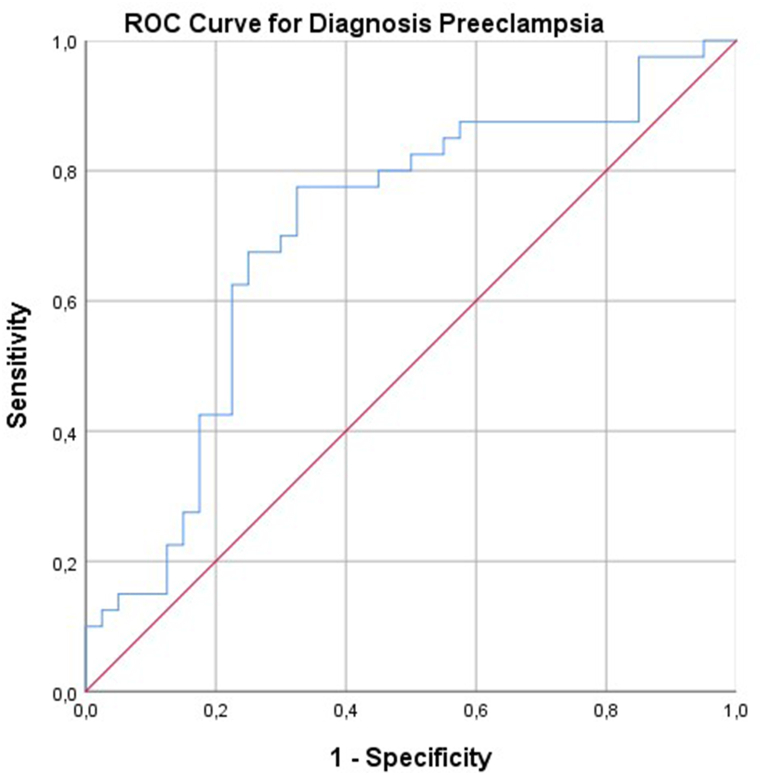
ROC analysis of SIII mean values in predicting the development of preeclampsia.

ROC analyses were performed to determine the sensitivity and specificity of NLR and SIII according to cut-off values in predicting preeclampsia development. The sensitivity and specificity for the cut-off value of 758.39 × 10^9^/L SIII in pregnant patients with preeclampsia was 77.5% and 67.5%, respectively (AUC: 0.705; 95% CI: 0.587–0.823; **p = 0.002**). No significant difference was noted between the mean NLR values in the diagnosis of preeclampsia. SIII was found to be a successful index in predicting the development of preeclampsia (Table 2 and Fig. 1).

**Table 2:** ROC analysis results of SIII values in the diagnosis of preeclampsia.

Parameter	Cut-off Value	Sensitivity	Specificity	Area under the curve (AUC)	95% CI		p
					Lower Bound	Upper Bound	
SIII ( × 10^9^/L)	758.39	77.5	67.5	0.705	0.587	0.823	**0.002**

In the model created using laboratory data to predict the development of preeclampsia, the increase in neutrophils and lymphocytes revealed that patients progressed to preeclampsia, while this was seen in the case of a decrease in platelet levels. In the logistic regression analysis performed in another modeling, NLR and SIII values revealed through laboratory data demonstrated that an increase in NLR and a decrease in SIII were crucial to indicate the progression to preeclampsia. In modeling with vital signs, an increase in diastolic blood pressure and a decrease in saturation and SIII are risk factors for preeclampsia (Table 3).

**Table 3:** Results of the logistic regression analysis of the parameters indicating progression to preeclampsia.

Parameter	Odd Ratio	B	p	95% CI
**Model 1**				
Neutrophil	1.286	0.252	**0.002**	1.097–1.507
Lymphocyte	3.656	1.296	**<0.001**	1.902–7.029
Platelet	0.986	−0.015	**0.006**	0.975–0.996
**Model 2**				
NLR	1.532	0.427	**0.017**	1.078–2.176
SIII	0.998	−0.002	**0.005**	0.996–0.999
**Model 3**				
SBP	−1.526	−0.004	**0.131**	−0.010–0.001
DBP	6.236	0.027	**<0.001**	0.019–0.036
Saturation	−2.952	−0.091	**0.004**	−0.152–(−0.029)
SIII	−0.817	−7.49	**0.038**	−9.25–(−3.12)
NLR	0.649	0.015	**0.518**	−0.031–0.060

## 4 Discussion

This study was conducted with 40 pregnant women diagnosed with preeclampsia and 40 pregnant patients who had hypertension during pregnancy but did not develop preeclampsia. SIII values were found to be lower in patients who developed preeclampsia. SIII is a cheap, easily calculable, objective parameter that allows evaluating both inflammation and platelet count. In this study, we have shown that SIII is a useful marker in the diagnosis of preeclampsia in pregnant patients with high blood pressure in the emergency department, considering that this marker enables simultaneous evaluation of the inflammatory process--which is crucial important for preeclampsia-- and platelet count, i.e., an indicator of coagulopathy. We think that SIII is a parameter that can predict the development of preeclampsia in hypertensive pregnant women.

Preeclampsia is one of the serious complications of pregnancy and its accurate diagnosis is of paramount importance. To the best of our knowledge, no biomarker currently exists to establish an accurate diagnosis of preeclampsia, one of the leading causes of morbidity and mortality in pregnancy. The need for markers supporting the diagnosis of preeclampsia is progressively increasing. Recent studies have reported that SIII is both an accurate indicator of inflammation and a useful ratio that helps to predict the diagnosis and prognosis of many diseases [12,13].

Inflammatory response was reported to be an important process in preeclampsia. One of the markers of inflammation is WBC count and the relationship between preeclampsia and WBC count has been investigated in many studies [5,14,15]. Liao et al. reported that increased WBC and neutrophil count had high sensitivity in predicting preeclampsia development [16]. Additionally, in another study by Örgülü et al. compared cases of early- and late-onset preeclampsia with other cases of women with healthy pregnancies, and they reported that WBC and neutrophil counts were higher in the cases of early- and late-onset preeclampsia [17]. Furthermore, in their review study, Campbell et al. reported that the number of B and T lymphocytes increased as a result of placental ischemia developing in patients with preeclampsia [18]. Similar to that reported in the literature, the WBC, neutrophil, and lymphocyte counts observed in the present study were higher in the preeclampsia group.

However, the results of the present study revealed no significant intergroup difference in terms of the NLR results. A review of literature showed that several studies that examined the relationship between preeclampsia and NLR have reported different results. In some studies, the NLR value was higher in patients with preeclampsia [5,19]. In another study, Cui et al. reported that NLR had a negative correlation with the diagnosis of preeclampsia owing to the increase in the number of lymphocytes in pregnant women with preeclampsia [20]. Yucel et al. compared NLR in pregnant women with preeclampsia and women with healthy pregnancy; they reported no significant intergroup difference and the results were similar to those observed in the present study [21].

The mechanism of thrombocytopenia in preeclampsia syndrome may be increased platelet consumption combined with increased megakaryocyte activity to compensate. Adhesion of platelets to damaged vascular endothelial areas leads to secondary destruction of platelets [22]. In their review study with 4892 women with preeclampsia, Woldeamanuel et al. reported that platelet count decreased in cases of preeclampsia and the platelet count may be considered a parameter that could diagnose preeclampsia [23]. Similarly, platelet count was lower in the preeclampsia group in our study. When the literature is examined, many studies investigating platelet counts of patients with preeclampsia have compared patients with preeclampsia with women with normal pregnancy. In the present study, we compared hypertensive patients with and without preeclampsia. Therefore, we think that the change in platelet count is not related to hypertension but endothelial damage is the main factor in this process.

SIII is used to assess the degree of systemic inflammation. Reportedly, SIII can be used to predict mortality in cancer patients. Li et al. suggested that high SIII levels may increase overall mortality and cardiovascular disease mortality in the overall population [24]. Another study by Yang et al. showed that high SIII values were associated with mortality from cardiovascular disease in hypertensive patients [25]. In a study by Cevher Akdulum et al., comparing pregnant patients diagnosed with preeclampsia with healthy pregnant women, SIII was observed to be low, similar to our study. However, we did not find any study in the literature comparing hypertensive pregnant patients with patients diagnosed with preeclampsia [26].

Our study results showed that SIII was significantly lower in the preeclampsia group than in the control group. The low SIII values in the preeclampsia group may be attributable to decreased platelet counts and increased lymphocyte counts. In our study, sensitivity + specificity was found to be 1.45. Additionally, it is recommended that the AUC be over 70% when using this biomarker, and in our study the AUC for SIII was found to be 0.705. In order to generalize the results of our study, studies with more patients are needed. We think that this rate will increase even more as the number of samples increases.

SIII enables the simultaneous assessment of both the inflammatory process and platelet values, which is an indicator of coagulopathy. Therefore, SIII may be considered a useful marker for the diagnosis of preeclampsia and in the follow-up of disease prognosis.

The biggest limitation of the study is its retrospective design. The role of this index in predicting preeclampsia can be revealed more clearly with prospective studies with larger samples.

## 5 Conclusion

In this study, we showed that SIII may be a marker that can guide clinicians in establishing the diagnosis of preeclampsia. We think that SIII is a critically important prognostic indicator in the diagnosis of preeclampsia because it simultaneously evaluates both neutrophil and lymphocyte values, which indicate the inflammation process, and platelet count, which is an indicator of coagulopathy. It is an easily calculable, objective, and low-cost marker, both because it is easy to calculate and because it can be calculated only with the hemogram result.

## Limitations

The limitations of our study were that it was a single-center and retrospective study. Additionally, the sample size of our study is small. In order to generalize the results of our study, it should be supported by multicenter, prospective studies with more cases.

## Funding declaration

This research did not receive any specific grant from funding agencies in the public, commercial, or not-for-profit sectors.

## Availability of data and materials

Data and materials are reachable from hospital automation information systems.

## Informed consent

Informed consent was obtained from all individual participants included in the study.

## Ethical approval

The ethics committee of the Basaksehir Cam and Sakura City Training and Research Hospital approved the study.

## Human rights

This manuscript was carried out in accordance with the Declaration of Helsinki and Good Clinical Practice guidelines.

## Ethical statement

**Compliance with Ethical Standards:** The study was performed according to the recommendations set by the Declaration of Helsinki on Medical Research involving Human Subjects.

**(in case of** Funding**)** Funding**:** This research received no specific grant from any funding agency in the public, commercial, or not-for-profit sectors.

## Ethical approval

This study was initiated after obtaining approval from the ethics committee (Ethics committee no: KAEK/2023.06.980).

## Informed consent

Since our study was planned retrospectively, an informed consent form was not required.

## CRediT authorship contribution statement

**Mucahit Kapci:** Writing – review & editing, Writing – original draft, Visualization, Supervision, Methodology, Investigation, Formal analysis, Data curation, Conceptualization. **Kemal Sener:** Writing – original draft, Methodology, Investigation, Formal analysis, Data curation, Conceptualization. **Ertugrul Altug:** Writing – original draft, Methodology, Investigation, Formal analysis, Data curation, Conceptualization, Writing – original draft, Methodology, Investigation, Formal analysis, Data curation, Conceptualization. **Akkan Avci:** Writing – review & editing, Writing – original draft, Supervision, Methodology, Investigation, Formal analysis, Writing – review & editing, Writing – original draft, Visualization, Supervision, Methodology, Investigation, Formal analysis, Data curation, Conceptualization.

## Declaration of competing interest

The authors declare that they have no known competing financial interests or personal relationships that could have appeared to influence the work reported in this paper.
